# Relative Quantification of Protein-Protein Interactions Using a Dual Luciferase Reporter Pull-Down Assay System

**DOI:** 10.1371/journal.pone.0026414

**Published:** 2011-10-19

**Authors:** Shuaizheng Jia, Jianchun Peng, Bo Gao, Zhongbin Chen, Yong Zhou, Qiuxia Fu, Haiping Wang, Linsheng Zhan

**Affiliations:** 1 Beijing Institute of Transfusion Medicine, Beijing, China; 2 Beijing Institute of Radiation Medicine, Beijing, China; 3 Department of Blood Transfusion, Hospital 307 of Chinese People's Liberation Army, Beijing, China; University of Texas Health Science Center at Houston, United States of America

## Abstract

The identification and quantitative analysis of protein-protein interactions are essential to the functional characterization of proteins in the post-proteomics era. The methods currently available are generally time-consuming, technically complicated, insensitive and/or semi-quantitative. The lack of simple, sensitive approaches to precisely quantify protein-protein interactions still prevents our understanding of the functions of many proteins. Here, we develop a novel dual luciferase reporter pull-down assay by combining a biotinylated Firefly luciferase pull-down assay with a dual luciferase reporter assay. The biotinylated Firefly luciferase-tagged protein enables rapid and efficient isolation of a putative Renilla luciferase-tagged binding protein from a relatively small amount of sample. Both of these proteins can be quantitatively detected using the dual luciferase reporter assay system. Protein-protein interactions, including Fos-Jun located in the nucleus; MAVS-TRAF3 in cytoplasm; inducible IRF3 dimerization; viral protein-regulated interactions, such as MAVS-MAVS and MAVS-TRAF3; IRF3 dimerization; and protein interaction domain mapping, are studied using this novel assay system. Herein, we demonstrate that this dual luciferase reporter pull-down assay enables the quantification of the relative amounts of interacting proteins that bind to streptavidin-coupled beads for protein purification. This study provides a simple, rapid, sensitive, and efficient approach to identify and quantify relative protein-protein interactions. Importantly, the dual luciferase reporter pull-down method will facilitate the functional determination of proteins.

## Introduction

Physical protein-protein interactions (PPIs) constitute a major mechanism for the regulation of many essential cellular and immunological functions, making PPIs essential components of biological systems. The high specificity and sensitivity of biological regulatory mechanisms depend on selective and dynamic PPI-mediated cellular responses to different stimuli. The reactions of cellular PPIs to environmental stimuli are essential to the host. However, aberrations in the patterns of PPIs for specific functions usually result in diseases. For example, chronic infection with hepatitis C virus (HCV) results from reduction of the dimerization of mitochondrial antiviral signaling protein (MAVS) by HCV nonstructural (NS) protein NS3/4A protease to levels that are too low to mount strong enough antiviral immune responses [1], [2]. Thus, measuring PPIs involved in a specific cellular compartment can shed light on how proteins work cooperatively in a cell.

Many methods have been developed to identify PPIs, including biophysical, biochemical and genetic approaches [3]. Traditional assays, such as co-immunoprecipitation and pull-down assays, which require the expression, purification of a fusion protein and Western blot, are technically complicated, time-consuming, costly and non-quantitative. Other methods also enable the monitoring of in vitro and in vivo PPIs, such as yeast two hybrid, fluorescence resonance energy transfer, bioluminescence resonance energy transfer, tandem affinity purification, mammalian protein-protein interaction trap and various protein complement assays that have recently been developed [4], [5]. The combined use of these approaches results in the identification of thousands of potential protein interactions. However, this process is generally time-consuming, technically complicated, insensitive and/or semi-quantitative. The lack of simple, sensitive approaches for the analysis of PPIs still hinders our understanding of many biological processes. Therefore, novel strategies are still needed to precisely characterize the components of protein complexes in the post-proteomics era.

Luminescence-based PPIs assays, such as luminescence-based mammalian interactome mapping (LUMIER) and the luminescence-based MBP pull-down interaction screening system (LuMPIS), only provide quantitative information about a Rluc-tagged protein among interacting protein pairs [6], [7]. Fluc and Rluc are regarded as dual luciferase reporters (DLRs), which are commonly combined to analyze relative protein expression levels. This DLR assay system provides a simple, rapid, sensitive, and quantitative means for the sequential measurement of Fluc and Rluc activities within a single sample [8]. Herein, we demonstrate that DLRs can be used in the analysis of PPIs and provide additional quantitative information about the relative amounts of interacting proteins that bind to beads in pull-down assays.

## Results

### Design and feasibility of the dual luciferase reporter pull-down assay

**Figure 1:**
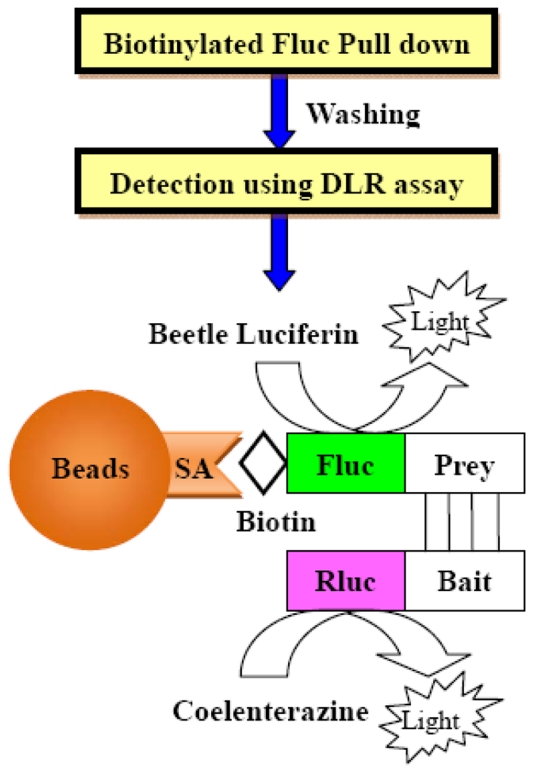
Design of the DLR-PD assay for the quantitative analysis of PPIs. Schematic diagram of the DLR-PD assay. Quantitative PPIs can be measured after the pull down of biotinylated Fluc-tagged prey proteins using SA-PMPs and bioluminescence detection of the biotinylated Fluc-tagged prey and Rluc-tagged bait proteins bound to the beads, respectively. SA, Streptavidin.

For the easy and efficient analysis of PPIs, we designed a novel dual luciferase reporter pull-down (DLR-PD) assay by combining the biotinylated Fluc pull-down assay with the DLR assay system (**Fig. 1**). A biotinylated protein pull-down assay, based on specific biotin-streptavidin interactions, is a small-scale affinity purification technique. The binding of biotin to streptavidin is the strongest noncovalent interaction known in nature. The high affinity of biotin for streptavidin allows for the simple, efficient, one-step purification of biotinylated proteins under high-stringency conditions [9].

**Figure 2:**
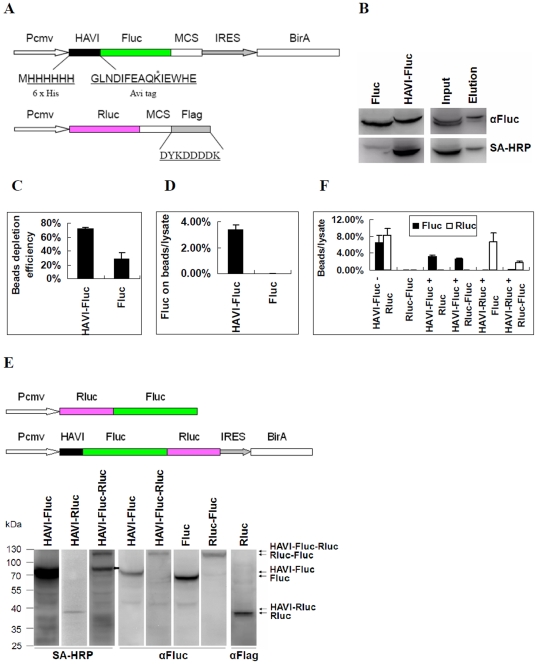
Feasibility of the DLR-PD assay. (**A**) Scheme for biotinylating the Fluc fusion protein by BirA biotin ligase and Rluc fusion protein in **Fig. 1**. The sequence of 6xHis and 15-aa Avi tag fused to the N-terminus of Fluc is shown. The asterisk indicates the lysine residue that is specifically biotinylated by BirA. The sequence of the Flag tag fused to the C-terminus of Rluc is shown. MCS, multiple clone sites; Pcmv, human cytomegalovirus (CMV) immediate-early gene promoter; IRES, internal ribosomal entry site. (**B**) Western blot analysis for the expression of biotinylated Fluc in HEK293 cells transfected with either a HAVI-Fluc or Fluc expression vector (Left). Biotinylated Fluc was detected by Western blot after a pull-down assay was performed on the cell lysate of HEK293 cells transfected with HAVI-Fluc and Fluc (Right). A three-fold addition of the cell lysate and SA-PMPs (100 µl) were used in the pull-down assay. The protein was eluted by boiling the beads after binding. The same membrane was probed with either anti-Fluc antibodies or streptavidin conjugated horseradish peroxidase (SA-HRP). (**C**) The bead depletion efficiency of Fluc indicates the depleted bead Fluc activity ratio compared with the lysate. This value was calculated by dividing depleted Fluc activity of the beads by the Fluc activity of the same amount of lysate used for the pull down. HEK293 cells were transfected with HAVI-Fluc or Fluc expression vectors. A pull-down assay was performed using SA-PMPs, and the Fluc activity of the lysate and the activity of the remaining Fluc in the lysate after pull down were measured. The depleted Fluc activity of the beads was equal to the Fluc activity of lysate minus the activity of the remaining Fluc of the lysate after the binding to the beads. (**D**) Percentage of Fluc on the beads compared with the lysate after pull down using SA-PMPs. This value was calculated by dividing Fluc activity measured on the beads by the Fluc activity in the same amount of lysate used for pull down. HEK293 cells were transfected with HAVI-Fluc or Fluc expression vectors. The pull-down assay was performed using SA-PMPs, and the Fluc activity on the beads and in the cell lysate was measured. (**E**) Schematic diagram of Rluc-Fluc and HAVI-Fluc-Rluc fusion expression constructs (Upper). Western blots of assayed protein expression in HEK293 cells transfected with HAVI-Fluc, HAVI-Rluc, HAVI-Fluc-Rluc, Fluc, Rluc-Fluc and Rluc-Flag expression vectors. The thick arrow indicates the endogenous biotinylated proteins (Down). SA-HRP, streptavidin conjugated horseradish peroxidase. (**F**) HEK293 cells were transfected or co-transfected with the indicated expression vectors. The pull-down assay was performed using SA-PMPs, and the Fluc and Rluc activity on the beads and in the cell lysate was measured. Beads/lysate indicates that the Fluc or Rluc activity ratio of the beads to the lysate. This value was calculated by dividing the Fluc (or Rluc) activity measured on the beads by the Fluc (or Rluc) activity in the same amount of lysate for the pull down.

To obtain biotinylated Fluc for the DLR-PD assay, Fluc with an HAVI tag sequence containing both 6 x His and Avi tags was coexpressed with bacterial biotin ligase enzyme BirA to covalently attach biotin to a specific lysine residue of the Avi Tag [10] (**Fig. 2A**). After the expression of HAVI-tagged Fluc in HEK293 cells, the biotinylation of this protein was determined by both Western blot and pull-down assays. As shown in **Fig. 2B**, HAVI-tagged Fluc was biotinylated, whereas Fluc was not. There was a small amount of unbiotinylated Fluc on the pull-down beads, representing nonspecific binding. The activity of the remaining Fluc in the cell lysate was quantitatively measured using the DLR assay after purification with Streptavidin MagneSphere Paramagnetic Particles (SA-PMPs). The Fluc activity of biotinylated HAVI-Fluc and nonbiotinylated Fluc were approximately 75% and 25% depleted, respectively (**Fig. 2C**). These data suggest that at least 50% of HAVI-Fluc was specifically bound to the beads if the nonspecific binding of HAVI-Fluc and Fluc are equal. However, when Fluc activity was directly measured on beads, only approximately 3.5% of the Fluc activity of HAVI-Fluc was detected on the beads (**Fig. 2D**). Notably, the nonspecific binding of unbiotinylated Fluc was significantly reduced after washing when Fluc activity was directly measured on beads (**Fig. 2D**). Therefore, Fluc activity was directly measured on beads instead of using the supernatant in subsequent experiments. These results suggest that biotinylated HAVI-Fluc fusion proteins can bind specifically to SA-PMPs beads. Importantly, the Fluc activity of the binding proteins can be quantitatively determined. To attenuate the effect of transfection variations on the amount of Fluc or Rluc bound to beads, the percentage of Fluc or Rluc activity of beads to cell lysate was used in all the experiments, which equals Fluc or Rluc activity on the beads divided by the Fluc or Rluc activity of the same amount of cell lysate used in the pull-down assays.

The lack of interactions between Fluc and Rluc is one of the most important prerequisites for the DLR-PD assay to work. To determine whether Fluc interacted with Rluc, the activity of both Rluc and Fluc on SA-PMP beads was measured. Rluc did not bind to co-expressed HAVI-Fluc after the pull-down assay was performed, nor did Fluc bind to HAVI-Rluc (**Fig. 2E,2F**). Moreover, the Rluc-Fluc fusion protein was observed to not bind HAVI-Fluc or HAVI-Rluc (**Fig. 2F**). These results indicate that there are no interactions between Fluc and Rluc. Furthermore, neither Fluc nor Rluc form dimers, making the DLR-PD assay theoretically feasible.

### Establishment of the DLR-PD assay using the Jun-Fos protein pair

**Figure 3:**
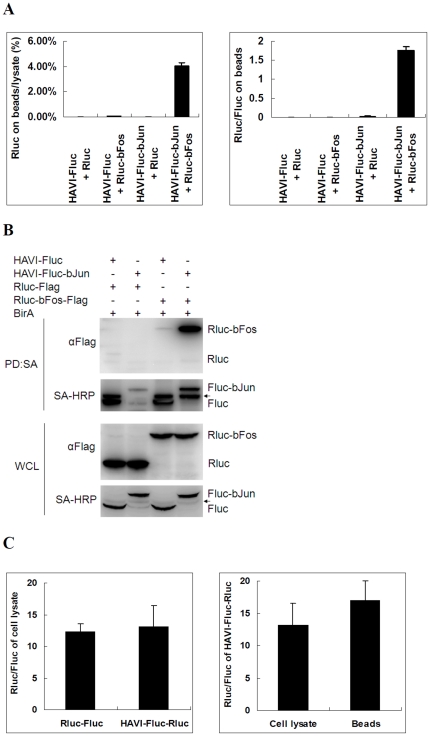
Analysis of bFos-bJun interactions in HEK293 cells. (**A**) HAVI-Fluc-bJun associated with Rluc-bFos when co-expressed in HEK293 cells using the DLR-PD assay. HEK293 cells were co-transfected with the indicated expression vectors (100 ng), and the DLR-PD assay was performed. The percentage of Rluc on the beads compared with the lysate was calculated by dividing the Rluc activity on the beads by the Rluc activity in the same amount of lysate used in the pull-down assay. The Rluc/Fluc ratio on the beads was calculated by dividing the Rluc activity by the Fluc activity measured on the beads. (**B**) HAVI-Fluc-bJun association with Rluc-bFos when co-expressed in HEK293 cells using the biotinylated protein pull-down assay. The arrow indicates endogenous biotinylated proteins. PD, pull down; SA, streptavidin; WCL, whole cell lysate. (**C**) The Rluc/Fluc activity ratio of Rluc-Fluc and HAVI-Fluc-Rluc in HEK293 cell lysate (Left). The Rluc/Fluc activity ratio of HAVI-Fluc-Rluc in the cell lysate and on the beads (Right). HEK293 cells were transfected with Rluc-Fluc and HAVI-Fluc-Rluc plasmids (100 ng), and cells were collected at 48 h post-transfection.

Human Jun and Fos form a heterodimeric transcription factor. Notably, these proteins strongly interact in the cellular nucleus *via* their basic leucine zipper regions, making human Jun and Fos classic model protein pairs for the development of novel PPI assays [7], [11]. To establish DLR-PD assays for the analysis of PPIs, biotinylated Fluc tag and Rluc tag were fused to the basic leucine zipper regions of Jun and Fos, respectively, through a flexible linker 4x(G_4_S). When the DLR-PD assay was carried out to analyze the interaction between Jun and Fos, the percentage of Rluc on beads to the HAVI-Fluc-bJun and co-expressed Rluc-bFos lysate used to identify PPIs was approximately 4.0% (**Fig. 3A**), which is more than 80-fold the percentage for the other three negative controls (P<0.05) and well above the cutoff value of 3-fold for positive PPIs in the original LUMIER assays using Rluc-tagged protein [6]. These data suggest that HAVI-Fluc-bJun interacts with Rluc-bFos, which is consistent with the results obtained from the traditional pull-down assay (**Fig. 3B**). Because bJun and bFos interact in the cellular nucleus, the DLR-PD assay is suitable for the analysis of PPIs in the nucleus [11].

In these experiments, the activity ratio of Rluc to Fluc (Rluc/Fluc) on the beads determined by the DLR-PD assay was used to indicate the relative amount of interacting proteins that bind to the beads. The activity ratio of Rluc/Fluc of Rluc-bFos to HAVI-Fluc-bJun on the beads is approximately 1.7∶1. To determine how the DLR-PD assay could be used to quantify the relative molar ratio of interacting proteins that bind to the beads based on the activity ratio of Rluc/Fluc on the beads, Rluc-Fluc and HAVI-Fluc-Rluc fusion molecules were used as controls. Although the molar ratio of Rluc/Fluc was 1∶1 for both proteins, the activity ratio of Rluc/Fluc of both proteins from the cell lysate was approximately 13∶1, and the activity ratio of Rluc/Fluc of HAVI-Fluc-Rluc on the beads was approximately 17∶1 (**Fig. 3C**). Therefore, the molar ratio of Rluc-bFos to HAVI-Fluc-bJun binding to the beads should be approximately 0.1∶1 when the activity ratio of Rluc/Fluc on the beads is approximately 1.7∶1(**Fig. 3A**). Thus, the DLR-PD assay allows us to determine the relative molar ratio of interacting proteins that bind to SA-PMP beads. However, this molar ratio only reflects the fusion proteins that bind to the beads and may not relate to the native Fos-Jun interaction, which presumably is 1∶1. Therefore, normalization using Rluc-Fluc and HAVI-Fluc-Rluc controls was not included in later experiments.

### Analysis of the dimerization of interferon regulation factor 3 using the DLR-PD assay

Quantitative analyses of PPIs in signaling pathways can contribute greatly to our understanding of protein functions and regulation mechanisms. Interferon (IFN) regulatory factor 3 (IRF3) is a master transcription factor for antiviral immune responses. In unstimulated cells, an IRF3 monomer is present in an inactive cytoplasmic form. Viral infection or other stimulation induces phosphorylation of IRF3 and the formation of IRF3 homodimers, leading to the activation of innate antiviral responses [12]. Therefore, IRF3 dimerization is one of the most important indicators of the activation of innate immune responses.

**Figure 4:**
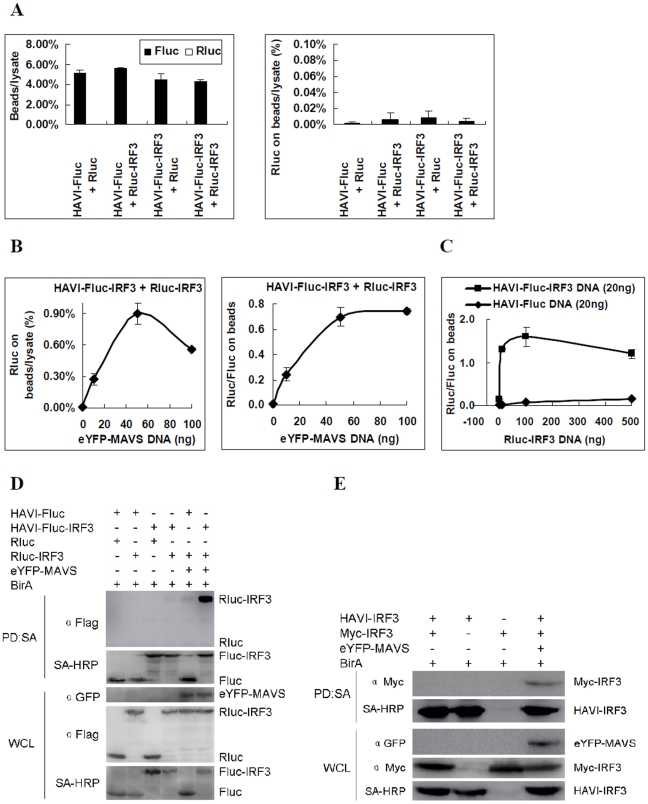
Analysis of IRF3 dimerization in the innate antiviral signaling pathway in HEK293 cells. (**A**) HAVI-Fluc-IRF3 did not associate with Rluc-IRF3 using the DLR-PD assay. HEK293 cells were co-transfected with the indicated expression vectors (100 ng), and the DLR-PD assay was performed. The Fluc and Rluc activity in lysate and on the beads were measured. The ratio of Fluc and Rluc activity on the beads compared with the cell lysate was calculated by dividing the Fluc and Rluc activity measured on the beads by the Fluc and Rluc activity of the same amount of lysate used in the pull-down assay. The percentage of Rluc on the beads compared with the cell lysate was calculated by dividing the Rluc activity measured on the beads by the Rluc activity of the same amount of lysate used in the pull-down assay. (**B**) HAVI-Fluc-IRF3 association with Rluc-IRF3 induced by overexpressed eYFP-MAVS in a dose-dependent manner using the DLR-PD assay. HEK293 cells were co-transfected with HAVI-Fluc-IRF3 (100 ng), Rluc-IRF3 (100 ng), and DNA (100 ng) containing eYFP-MAVS vector (0, 10, 50 or 100 ng) and control plasmid pcDNA3.1. The percentage of Rluc on the beads compared with the lysate was calculated by dividing the Rluc activity of Rluc-IRF3 measured on the beads by the Rluc activity in the same amount of lysate used in the pull-down assay. The Rluc/Fluc ratio on the beads was calculated by dividing the Rluc activity by the Fluc activity measured on the beads. (**C**) HAVI-Fluc-IRF3 association with Rluc-IRF3 induced by overexpressed eYFP-MAVS in HEK293 cells using the biotinylated protein pull-down assay. (**D**) Evaluation of the HAVI-IRF3 association with Myc-IRF3 induced by overexpressed eYFP-MAVS in HEK293 cells using the biotinylated protein pull-down assay. (**E**) The curve of the Rluc/Fluc ratio on the beads based on the DLR-PD assay results. HEK293 cells were transfected with eYFP-MAVS (50 ng), HAVI-Fluc-IRF3 (20 ng) or HAVI-Fluc (20 ng) and Rluc-IRF3 plasmid DNA (1, 10, 100 or 500 ng). Cells were collected at 48 h post-transfection and subsequently used in the DLR-PD assay.

To relatively quantify IRF3 dimerization via the DLR-PD assay, IRF3 was fused with a biotinylated Fluc tag and a Rluc tag. Even though HAVI-Fluc-IRF3 was co-overexpressed with Rluc-IRF3 in HEK293 cells, the percentage of Rluc on beads compared with the cell lysate was close to that of three negative controls (P>0.05)(**Fig. 4A**). These data suggest that IRF3 does not dimerize without stimulation.

In contrast, when both HAVI-Fluc-IRF3 and Rluc-IRF3 were co-overexpressed with MAVS, a critical upstream adaptor in IRF3 activation signaling, the amount of Rluc-IRF3 that bound to the beads increased in a MAVS dose-dependent manner after a pull-down assay of HAVI-Fluc-IRF3 with SA-PMPs was performed. The percentage of Rluc on the beads compared with the cell lysate increased approximately 27-fold when HEK293 cells were transfected with only 10 ng of the MAVS vector compared with the negative control (P<0.05) (**Fig. 4B**). This percentage is much higher than the cutoff value of 3-fold for positive PPIs [6]. Therefore, the dimerization of IRF3, which was stimulated with a small amount of the MAVS expression vector, was observed using the DLR-PD assay. The activity ratio of Rluc/Fluc in interacting Rluc-IRF3 and HAVI-Fluc-IRF3 on the beads increased 75-fold, from 0.01∶1 to 0.75∶1 when the amount of transfected MAVS increased from 10 ng to 100 ng (P<0.05) (**Fig. 4B**). The formation of a saturation curve based on the transfection of MAVS in HEK293 cells suggested that overexpressed MAVS induced IRF3 dimerization. When HAVI-Fluc-IRF3 or HAVI-Fluc was co-transfected with MAVS with an increasing amount of Rluc-IRF3 in HEK293 cells, the Rluc/Fluc ratio on the beads of HAVI-Fluc-IRF3 became saturated very quickly, which was much higher than that of HAVI-Fluc (**Fig. 4C**). These observations confirmed that Rluc-IRF3 interacts specifically with HAVI-Fluc-IRF3, but not with HAVI-Fluc.

To validate the DLR-PD data, a pull-down assay detected by a traditional Western blot was also performed. Rluc-IRF3 interacted with HAVI-Fluc-IRF3 when HEK293 cells were co-transfected with MAVS, but these proteins did not interact with each other without the transfection of MAVS (**Fig. 4D**). Similarly, HAVI-IRF3 interacting with Myc-IRF3 was also observed only after stimulation with MAVS, as evidenced by the pull-down assay (**Fig. 4E**). These results suggest that the DLR-PD assay enables the identification and relative quantification of IRF3 dimerization.

**Figure 5:**
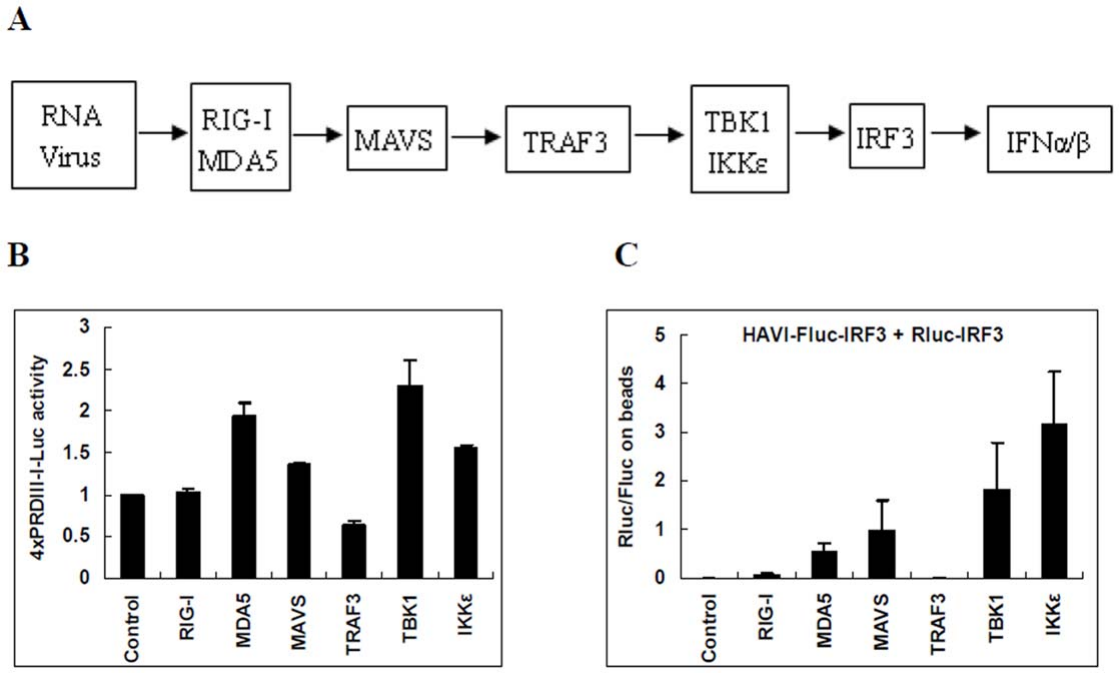
Comparison of IRF3 dimerization using the DLR-PD assay and activation of the IRF3 regulatory element PRDIII-I induced by different molecules in HEK293 cells. (**A**) Schematic diagram of virus-activated innate antiviral signaling pathways. (**B**) Activation of the IRF3 regulatory element PRDIII-I induced by different expression vectors (control pCDNA3.1, GFP2-RIG-I, Halo-MDA5, eYFP-MAVS, Myc-TRAF3, Myc-TBK1 or Myc-IKKε) in HEK293 cells 24 h post-transfection. The IRF3 regulatory element PRDIII-I was assayed with 4xPRDIII-I-Fluc and the pRL-TK dual reporter. After being normalized by Rluc activity, Fluc activity from different cell lysates was compared with that of control pCDNA3.1-transfected cells. (**C**) Analysis of the association of HAVI-Fluc-IRF3 with Rluc-IRF3 induced by different molecules using the DLR-PD assay. HEK293 cells were co-transfected with HAVI-Fluc-IRF3 (20 ng), Rluc-IRF3 (10 ng) and different expression vectors (100 ng of control pCDNA3.1, GFP2-RIG-I, Halo-MDA5, eYFP-MAVS, Myc-TRAF3, Myc-TBK1 or Myc-IKKε). Subsequently, the DLR-PD assay was performed. The Rluc/Fluc ratio on the beads was calculated by dividing the Rluc activity by the Fluc activity measured on the beads.

IRF3 dimerization is an important indicator of IRF3 activation. The assay to measure the activity of IRF3 regulatory element PRDIII-I regulated reporter induced by the over-expression of different molecules in the cytoplasmic antiviral signal pathway [13], [14] (**Fig. 5A**) indicated that MDA5, MAVS, TBK1, and IKKε induced approximately only a 2-fold IRF3 reporter activation in HEK293 cells (**Fig. 5B**). In contrast, using the established DLR-PD assay, the IRF3 dimerization ratio increased up to a thousand-fold when some molecules, such as IKKε, were overexpressed (**Fig. 5C**). Although the DLR-PD assay indicates IRF3 dimer formation and the 4xPRDIII-I luciferase activity reflects the activation of endogenous IRF3[15], the results of both assays are consistent when monitoring IRF3 activation.

### Using the DLR-PD assay to analyze MAVS dimerization and inhibition by HCV NS3/4A

**Figure 6:**
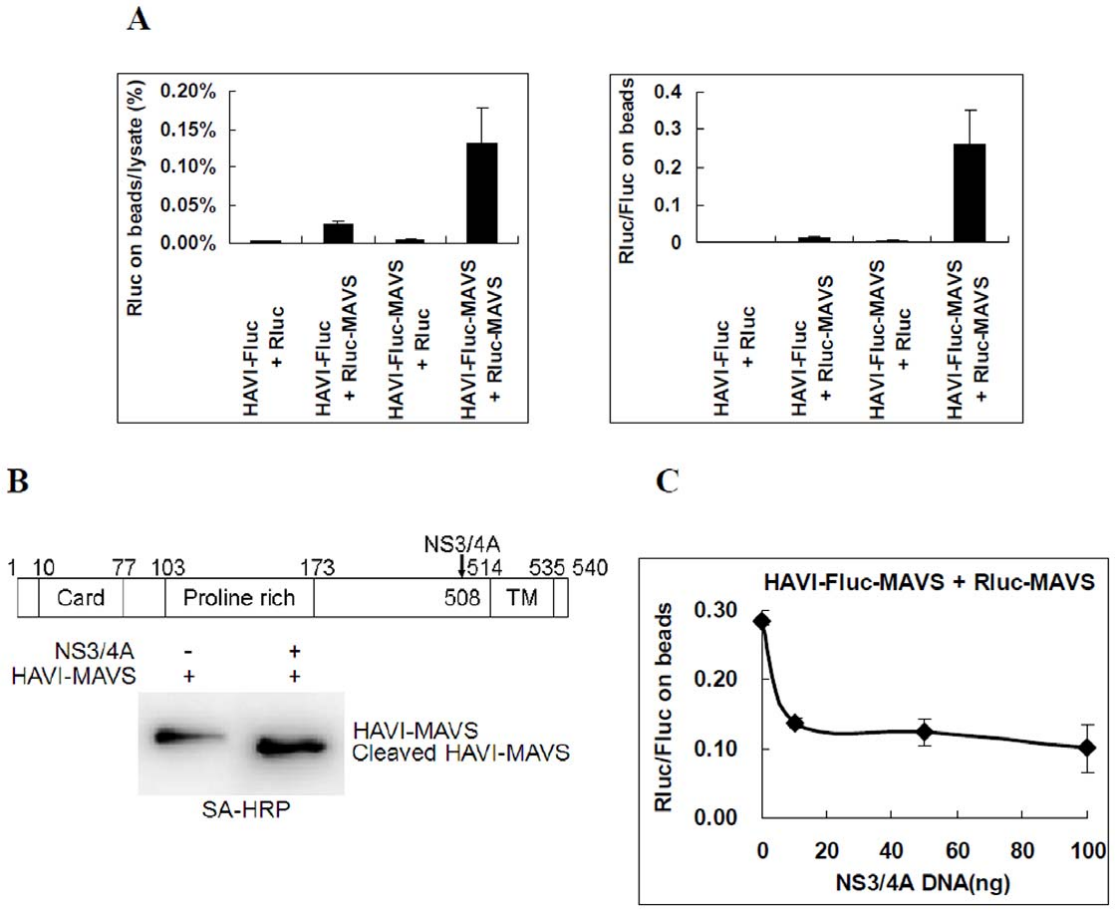
Analysis of MAVS dimerization and inhibition by HCV NS3/4A in HEK293 cells. (**A**) Evaluation of the HAVI-Fluc-MAVS association with Rluc-MAVS using the DLR-PD assay. HEK293 cells were co-transfected with the indicated vectors (100 ng). The percentage of Rluc on the beads compared with the cell lysate was calculated by dividing the Rluc activity of Rluc-MAVS on the beads with the Rluc activity in the same amount of lysate used in the pull-down assays. The Rluc/Fluc ratio on the beads was calculated by dividing the Rluc activity by the Fluc activity measured on the beads. (**B**) HAVI-MAVS was cleaved by HCV NS3/4A and detected by Western blot when coexpressed in HEK293 cells. (**C**) Analysis of the interactions between HAVI-Fluc-MAVS and Rluc-MAVS inhibited by HCV NS3/4A using the DLR-PD assay. HEK293 cells were co-transfected with HAVI-Fluc-MAVS (100 ng), Rluc-MAVS (100 ng) and DNA (100 ng) containing the NS3/4A vector (0, 10, 50 or 100 ng) and control plasmid pCI neo. The Rluc/Fluc ratio on the beads was calculated by dividing the Rluc activity by the Fluc activity measured on the beads.

To determine whether the DLR-PD assay is applicable to the analysis of membranous protein interactions, MAVS dimerization was measured using the DLR-PD assay. MAVS is located on the outer membrane of mitochondria, and its self-association mediates antiviral innate immune signaling [16], [17]. When HAVI-Fluc-MAVS was co-overexpressed with Rluc-MAVS in HEK293 cells, the percentage of Rluc on the beads compared with the cell lysate was 0.13%, which is at least 6-fold higher than that of three negative controls (P<0.05) and higher than the positive PPI cutoff value of 3-fold [6] (**Fig. 6A**). These data suggest that HAVI-Fluc-MAVS interacted with Rluc-MAVS when they were co-expressed, confirming the dimerization of MAVS. Moreover, the activity ratio of Rluc/Fluc of Rluc-MAVS and HAVI-Fluc-MAVS on the beads was approximately 0.28∶1(**Fig. 6A**), providing a relative amount of interacting HAVI-Fluc-MAVS and Rluc-MAVS. These results indicate that the DLR-PD assay is able to quantitatively measure the PPIs of membranous protein.

HCV NS3/4A has been shown to cleave MAVS, blocking the dimerization of MAVS, which has been shown to be critical for innate antiviral responses and cleavage-induced redistribution of MAVS cellular localization [2], [15]. We also proved that HCV NS3/4A cleaved MAVS in HEK293 cells (**Fig. 6B**). To quantitatively determine how HCV NS3/4A regulated the dimerization of MAVS, HAVI-Fluc-MAVS, Rluc-MAVS and NS3/4A were co-expressed in HEK293 cells, and the dimerization was measured using the DLR-PD assay. The results showed that the activity ratio of Rluc/Fluc in interacting Rluc-MAVS and HAVI-Fluc-MAVS on the beads decreased in an HCV NS3/4A dose-dependent manner (**Fig. 6C**). Therefore, HCV NS3/4A inhibited MAVS dimerization, which is consistent with previous studies [2], [16], [18].

### Analysis of MAVS-TRAF3 interactions and regulation by HCV NS3/4A using the DLR-PD assay

**Figure 7:**
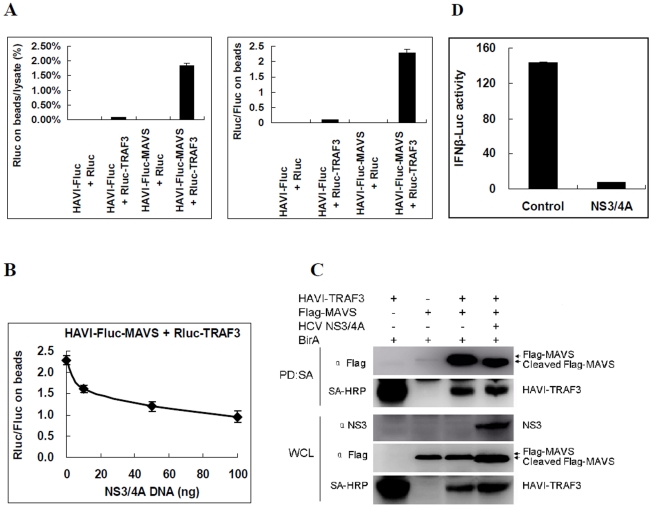
Analysis of MAVS-TRAF3 interactions and regulation by HCV NS3/4A in HEK293 cells. (**A**) HAVI-Fluc-MAVS was determined to be associated with Rluc-TRAF3 using the DLR-PD assay. HEK293 cells were co-transfected with the indicated vectors. The percentage of Rluc on the beads compared with the cell lysate was calculated by dividing the Rluc activity of Rluc-TRAF3 measured on the beads by the Rluc activity in the same amount of lysate used in the pull-down assay. The Rluc/Fluc ratio on the beads was calculated by dividing the Rluc activity by the Fluc activity measured on the beads. (**B**) Using the DLR-PD assay, the HAVI-Fluc-MAVS association with Rluc-TRAF3 was determined to be inhibited by HCV NS3/4A in a dose-dependent manner in HEK293 cells. Cells were co-transfected with HAVI-Fluc-MAVS (100 ng), Rluc-TRAF3 (100 ng) and DNA (100 ng) containing the NS3/4A vector (0, 10, 50 or 100 ng) and control plasmid pCI neo. The Rluc/Fluc ratio on the beads was calculated by dividing the Rluc activity by the Fluc activity measured on the beads. (**C**) Biotinylated protein pull-down assay to determine the MAVS-TRAF3 interactions and regulation by HCV NS3/4A in HEK293 cells. (**D**) Overexpression of HCV NS3/4A inhibited the MAVS-induced activation of the IFNβ-Luc promoter after normalization with RL-TK in HEK293 cells. The Fluc activity from different cell lysates was divided by that of cell lysates from mock transfected cells without MAVS activation.

TRAF3 has been shown to be a downstream molecule of MAVS in the RIG-I-MAVS cytoplasmic antiviral signal pathway. Specific interactions between MAVS-TRAF3 regulate the signaling of MAVS [19]. To quantitatively measure MAVS-TRAF3 interactions, HAVI-Fluc-MAVS was co-overexpressed with Rluc-TRAF3 in HEK293 cells, and the DLR-PD assay was performed. The percentage of Rluc on the beads compared with the cell lysate was 1.85% (**Fig. 7A**), which is more than 26-fold higher than that of three negative controls (P<0.05) and higher than the cutoff value of 3-fold for positive PPIs [6]. These results suggest that MAVS interacts with TRAF3. Additionally, interactions between MAVS and TRAF3 were also confirmed by a traditional pull-down assay (**Fig. 7C**). The results of the DLR-PD assay demonstrated that the activity ratio of Rluc/Fluc to Rluc-TRAF3 and HAVI-Fluc-MAVS on the beads was approximately 2.29∶1(**Fig. 7A**), indicating the relative amount of interacting proteins.

Previous studies have shown that HCV NS3/4A cleaved MAVS and inhibited the dimerization of MAVS [16], [18], but how it regulates MAVS-TRAF3 interactions remains unclear. To determine how HCV NS3/4A regulates these interactions, HCV NS3/4A was cotransfected with Rluc-TRAF3 and HAVI-Fluc-MAVS in HEK293 cells, and the DLR-PD assay was performed. The activity ratio of Rluc/Fluc to Rluc-TRAF3 and HAVI-Fluc-MAVS significantly decreased from 2.29∶1 to 0.956∶1 when the amount of transfected HCV NS3/4A DNA increased from 10 ng to 100 ng (**Fig. 7B**). These results were consistent with those from the traditional pull-down assay (**Fig. 7C**), suggesting that HCV NS3/4A resulted in a smaller amount of TRAF3 binding to the same amount of cleaved MAVS compared with full length of MAVS. Because the cleavage site of MAVS by HCV NS3/4A is close to its transmembrane (TM) domain, our results also suggested that the MAVS TM domain plays a very important role in MAVS-TRAF3 signaling (**Fig. 7C**). These results demonstrate that cleavage of MAVS by HCV NS3/4A not only inhibits the self-association of MAVS (**Fig. 6D**) but also blocks the recruitment of TRAF3 to MAVS (**Fig. 7C**). Therefore, HCV NS3/4A lowers the capability of MAVS to activate its downstream molecule TRAF3 through the cleavage of MAVS, which in turn results in the inhibition of innate antiviral responses (**Fig. 7D**).

### Application of the DLR-PD assay to protein interaction domain mapping

**Figure 8:**
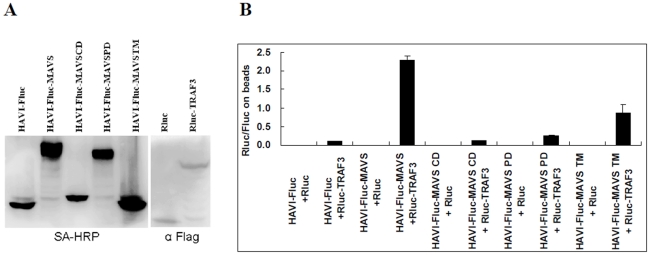
Application of the DLR-PD assay to the domain mapping of the interaction of MAVS with TRAF3. (**A**) Western blot of HAVI-Fluc-tagged MAVS domains and Rluc-tagged TRAF3 in HEK293 cells. (**B**) Domain mapping of the interaction of MAVS with TRAF3 using the DLR-PD assay. HEK293 cells were co-transfected with Rluc-TRAF3, and the different domains of MAVS were fused with HAVI-Fluc vectors. MAVS CD, MAVS Card domain; MAVS PD, MAVS proline-rich domain; MAVS TM, MAVS transmembrane domain. The Rluc/Fluc ratio on the beads was calculated by dividing the Rluc activity by the Fluc activity measured on the beads.

Protein interaction domain mapping is very important for studying PPIs. To determine whether the DLR-PD assay is suitable for domain mapping, the domain of MAVS, which is responsible for the interaction with TRAF3, was analyzed (**Fig. 8A**). The results indicated that the MAVS TM domain played a more important role than the proline-rich and CARD domains in MAVS-TRAF3 interactions (P<0.05) (**Fig. 8B**). However, the molecular integrity of MAVS was critical for MAVS and TRAF3 to interact, which is consistent with the result of the traditional pull-down assay (**Fig. 7C**).

## Discussion

In the present study, we describe a novel DLR-PD assay for the relative quantification of interacting proteins that bind to beads for pull down. The DLR-PD assay system is based on the combination of biotinylated Fluc pull-down and DLR assays. The biotinylated Fluc-tagged protein acts as the prey to capture a putative binding bait protein tagged with Rluc. Unlike a Western blot that is used for detecting the amount of interacting proteins in a typical pull-down assay, the DLR-PD assay is used to determine the amount of biotinylated Fluc-prey and Rluc-bait fusion protein on the beads and in the cell lysate by measuring the activities of Fluc and Rluc. This assay allows for the quantification of the relative amount of purified interacting protein pairs (**Fig. 3A** **,** **4B** **,** **6A** **, and** **7A**), which will be useful in the identification of a novel protein that regulates PPIs in a known signaling pathway, such as NS3/4A (**Fig. 6C** and **7C**). Moreover, through the measurement of the Fluc and Rluc activities of Fluc- and Rluc-tagged proteins in the cell lysate, the expression levels of tested proteins can be determined prior to performing the DLR-PD assay, and the results can be used as internal controls in the DLR-PD assay. In this case, the DLR-PD assay is superior to already available methods, such as two-hybrid or split-luciferase systems, for determining the expression levels of tested proteins. However, two-hybrid and split-luciferase systems enable us to measure protein-protein interactions in vivo non-invasively, which is not possible in DLR-PD assays.

Both LUMIER and LuMPIS assays also use Rluc-tagged proteins to determine PPIs [6], [7]. However, these assays only provide quantitative information about the Rluc-tagged protein among interacting protein pairs, similar to the results shown in the left of **Figs. 3A** **,** **4B** **,** **6A** and **7A**. Because Fluc-tagged proteins can be used to determine PPIs in DLR-PD assays, the relative amount of PPIs can be quantified as shown in the right of **Figs. 3A** **,** **4B** **,** **6A** and **7A**. Furthermore, because Fluc-tagged proteins can serve as internal controls to limit variations of the amount of prey proteins, the result regarding IRF3 dimer analysis in the right of **Fig. 4B** is more accurate than that in the left. As a result, DLR-PD assays provide more quantitative information about PPIs than the LUMIER and LuMPIS methods. DLR-PD assays even allow for the quantification of the molar ratio of purified interacting protein pairs if suitable controls, such as Rluc-Fluc or HAVI-Fluc-Rluc, are included (**Fig. 3C**). The relative molar ratio of interacting proteins determined by the DLR-PD assay only reflects interacting proteins that bind to the beads and may not relate to native PPIs. For example, the molar ratio of Rluc-bFos to HAVI-Fluc-bJun that bound to the beads was measured to be approximately 0.1∶1 based on our DLR-PD assay results (**Fig. 3A and 3C**). This molar ratio is different from the native Fos-Jun interaction, which presumably is 1∶1. Furthermore, the DLR-PD assay may not be suited to measure the affinity between interacting proteins, as is the case with both the LUMIER and LuMPIS methods [6], [7].

We observed that bound SA-PMPs depleted the activity of approximately 75% of biotinylated HAVI-Fluc when the Fluc activity value of the lysate after binding SA-PMPs was subtracted from the Fluc activity value of the lysate prior to binding (**Fig. 2C**). However, it was difficult to discriminate specific binding from nonspecific binding because approximately 25% of unbiotinylated Fluc was also bound to the beads. Therefore, the pull-down efficiency of our assay is at least 50% if nonspecific binding is also taken into consideration. When Fluc activity was directly measured on the beads after washing and divided by the Fluc activity of the cell lysate prior to binding, the nonspecific binding of unbiotinylated Fluc was significantly reduced (**Fig. 2D**). However, the percentage of beads bound to Fluc and Rluc compared with the cell lysate was only approximately 5% or even lower in most DLR-PD assays (**Figs. 2D** **,** **3A** **,** **4A** and **7A**). Because it is impossible to interrupt the binding between biotin and streptavidin by washing with PBS alone, we hypothesize that this low ratio is a result of the inhibition of the activity of Fluc and Rluc by the magnetic beads rather than low the pull-down efficiency of biotinylated proteins. Nevertheless, Fluc activity was directly measured on the beads in all experiments because nonspecific binding was significantly reduced.

It is important to use biotinylated Fluc-tagged prey proteins for capturing putative Rluc-tagged binding bait proteins. The ratio of Rluc/Fluc activity is approximately 12–14∶1 when the molar ratio of Rluc/Fluc is 1∶1 (**Fig. 3C**). In addition, the value of Rluc/Fluc increased when both Rluc and Fluc proteins bound to the beads compared with them being in solution (**Fig. 3C**). These two features increase the sensitivity of the DLR-PD assay for the quantitative analysis of PPIs, which will be especially helpful for the analysis of PPIs with a low ratio of Rluc/Fluc on the beads, such as the interactions of MAVS (**Fig. 6A**).

Regarding the analysis of dimer formation for IRF3, the ratio of Rluc to Fluc on the beads in **Fig. 4B** was extremely low. It is possible that two HAVI-Fluc-IRF3 molecules as well as two Rluc-IRF3 molecules interacted with each other, respectively, to form a dimer. The Rluc/Fluc ratio on the beads reached a maximum value and then slowly decreased when the amount of Rluc-IRF3 increased continuously, which may support the hypothesis that two Rluc-IRF3 molecules formed a dimer (**Fig. 4C**). The same is true for the analysis of MAVS dimerization in **Fig. 6A**. The experiment showing the dependence of the IRF3 interaction on an upstream regulator demonstrates that the new method is flexible and can be adapted to different protein interactions.

In summary, a novel biochemical DLR-PD assay was established for analysis of PPIs. Examples of nuclear PPIs (Fos-Jun), cytoplasmic membranous PPIs (MAVS-MAVS, MAVS-TRAF3), inducible PPIs (e.g., IRF3 dimerization induced by MAVS and TBK1), and viral protein-regulated PPIs (MAVS-MAVS, MAVS-TRAF3 by HCV NS3/4A) were studied using the DLR-PD assay. The applications of the DLR-PD assay to analyze PPIs, such as protein interaction domain mapping and viral protein targets in innate antiviral signaling pathways, were explored. The DLR-PD assay system was demonstrated to provide a simple, rapid, sensitive, and efficient approach to analysis of PPIs and will be useful for the functional determination of protein complexes.

## Materials and Methods

### Constructs

The coding sequences of genes were amplified by PCR using Platinum Pfx DNA polymerase (Invitrogen). The primers and other DNA sequences are listed in Table 1. The fragments were cloned using the pEASY-Blunt Cloning Kit (Transgen) and verified by sequencing. The EMCV IRES was amplified from the pIRES vector (Clontech). The coding region of the birA biotin-protein ligase gene was amplified from *E. coli* genomic DNA. EMCV IRES and the birA gene were ligated using the SacII restriction site. The EMCV IRES-driven birA gene was recloned into the SmaI and NotI restriction sites of the mammalian cell expression vector pCI neo (Promega). The HAVI tag sequence containing both 6 x HIS and the Avi tags for biotinylation and multiple clone sites were synthesized by the Augct company and cloned into the NheI and SmaI restriction sites of pCI neo with the EMCV IRES-driven birA gene to construct the biotinylation expression vector pHAVI. The Fluc gene was amplified from pGL3-Basic (Promega), digested with SalI and XhoI restriction enzymes and religated into the XhoI restriction site of pHAVI to construct pHAVI-Fluc. The fragment containing Rluc was amplified from pRL-TK (Promega), digested with XhoI and XbaI restriction enzymes and cloned into the XhoI and XbaI restriction sites of pHAVI to construct pHAVI-Rluc. The fragment containing Rluc digested with NheI and SmaI restriction enzymes was cloned into the XbaI and EcoRV restriction sites of pHAVI-Fluc to construct the pHAVI-Fluc-Rluc vector. A flag tag sequence was synthesized by the Augct Company and cloned into the SalI and NotI restriction sites of pCI neo to construct the pCI-Flag vector. The Rluc gene was ligated into the NheI and XhoI restriction sites of pCI-Flag to construct the pCI-Rluc-Flag vector. The pCI-Rluc-Fluc vector was formed by cloning the Fluc fragment with XhoI and SmaI restriction sites into the XhoI and EcoRV restriction sites of the pCI-Rluc-Flag vector. pCMV-Fluc was constructed by cloning the CMV promoter from pCI neo into the pGL3-Basis vector. The pRL-CMV vector was bought from the Promega Company. The NS3/4A sequence derived from pCon1-FL was cloned into the EcoRI and XbaI restriction sites of pCI neo. TRAF3, IRF3, and MAVS were cloned into the XhoI and MluI restriction sites of pHAVI to construct the pHAVI-TRAF3, pHAVI-IRF3, and pHAVI-MAVS vectors, respectively. IRF3, MAVS, MAVS Card (MAVS CD) and the proline-rich MAVS domain (MAVS PD) were cloned into the XhoI and MluI restriction sites of pHAVI-Fluc to construct the pHAVI-Fluc-IRF3, pHAVI-Fluc-MAVS, pHAVI-Fluc-MAVSCD, and pHAVI-Fluc-MAVSPD vectors, respectively. The MAVS TM was cloned into the MluI and SalI restriction sites of pHAVI-Fluc to construct the pHAVI-Fluc-MAVSTM vector. TRAF3, IRF3, and MAVS were cloned into the XhoI and MluI restriction sites of pCI-Rluc-Flag to express the Rluc-TRAF3-Flag, Rluc-IRF3-Flag and Rluc-MAVS-Flag proteins, respectively. The basic leucine zipper (bZIP) regions of Jun (225–334) and Fos (118–220) were synthesized by the Generay Company and cloned into the XhoI and MluI restriction sites of pHAVI-Fluc and pCI-Rluc-Flag to construct the pHAVI-Fluc-bJun and pCI-Rluc-bFos-Flag vectors, respectively. pIFNβ-Luc was constructed by subcloning the human IFNβ promoter (−281– +20) into pTAL-Luc (Clontech) between the NheI and EcoRI restriction sites. pHalo-MDA5 was obtained from GeneCopoeia Inc. pNFκB-Luc and pISRE-Luc were obtained from Clontech.

**Table 1:** Primers and other DNA sequences used in the experiment.

Name	Sequences (5′-3′)	Product size (bp)
BirA SacII F	GCCCGCGGATGAAGGATAACACCGTGCCA	980
BirA NotI R	GCGCGGCCGCTTATTTTTCTGCACTACGCA	
IRES SalI SmaI F	TCGTCGACTGACCCGGGAATTCCGCCCCTCTCCCTCCCCC	606
IRES SacII R	TCCCGCGGTTATCATCGTGTTTTTCAAAGGA	
NheI HAVI XhoI F	CTAGCATGCATCATCACCATCACCACGGCTTGAACGACATCTTCGAGGCCCAGAAGATCGAGTGGCACGAGC	
NheI HAVI XhoI R	TCGAGCTCGTGCCACTCGATCTTCTGGGCCTCGAAGATGTCGTTCAAGCCGTGGTGATGGTGATGATGCATG	
Fluc SalI F	TCGTCGACATGGAAGACGCCAAAAACATAAA	1678
Fluc XhoI R	CGCTCGAGAGATCCAGAGCCCACGGCGATCTTTCCGCCCTT	
Rluc NheI F	AG GCTAGCATGACTTCGAAAGTTTATGAT	951
Rluc XhoI R	AGCTCGAGTTGTTCATTTTTGAGAACTCGCT	
SalIFlagNotIF	TCGACGATTACAAGGACGATGACGATAAGTGA	
SalIFlagNotIR	GGCCGCTCACTTATCGTCATCGTCCTTGTAATCG	
MAVS XhoI F	GCCTCGAGATGCCGTTTGCTGAAGACAAGA	1636
MAVS MluI R	TCACGCGTGTGCAGACGCCGCCGGTACAGCA	
MAVS CARD MluI R	TCACGCGTACGGTCCGAGGTCCGAGGCT	322
MAVS PD XhoI F	GCCTCGAGATGCCCCCAGACCCACTGGAGCCA	1252
MAVS PD MluI R	TCACGCGTAGGTGAGGGCCTGTGGCATGG	
MluI - MAVSTM- SalI F	CGCGTGGGGCTCTGTGGCTCCAGGTGGCTGTGACAGGGGTGCTGGTAGTCACACTCCTGGTGGTGCTGTACG	
MluI- MAVSTM-Sal R	TCGACGTACAGCACCACCAGGAGTGTGACTACCAGCACCCCTGTCACAGCCACCTGGAGCCACAGAGCCCCA	
TRAF3 XhoI F	GCCTCGAGATGGAGTCGAGTAAAAAGATGG	1720
TRAF3 MluI R	TCACGCGTGGGATCGGGCAGATCCGAAG	
IRF3 XhoI F	GCCTCGAGATGGGAACCCCAAAGCCACGGA	1297
IRF3 MluI R	TCACGCGTGCTCTCCCCAGGGCCCTGGAAA	

### Reagents

Lipofectamine 2000 transfection reagent was purchased from Invitrogen. Streptavidin MagneSphere Paramagnetic Particles (SA-PMPs), the Dual-Luciferase Reporter Assay System and goat polyclonal antibody to Fluc (G7451) were all obtained from Promega. Anti-flag tagged mouse monoclonal antibodies (3B9) were obtained from Abmart. Anti-Myc tagged mouse monoclonal antibodies (9E10) were obtained from Beijing Protein Innovation Company. Anti-HCV NS3 mouse monoclonal antibodies were purchased from the Millipore Company. Anti-GFP rabbit polyclonal antibodies were bought from the Proteintech Group. Horseradish peroxidase (HRP) conjugated streptavidin (SA-HRP) was obtained from CWBio.

### DNA transfection

HEK293 cells were plated at approximately 2.5×10^5^ cell/well in 24-well plates and grown overnight to obtain 70–80% confluent monolayer cells. DNA plasmids (0.2–0.5 µg) were transfected using the Lipofectamine 2000 transfection reagent according to the manufacturer's protocol. Briefly, Opti-MEM I (50 µl) and Lipofectamine2000 (1.5 µl) were incubated for 5 m at room temperature. DNA (0.2–0.5 µg) was added to the mixture and incubated for an additional 20 m. After the medium was removed from the 24-well plate, the DNA mixture and Opti-MEM I (250 µl) were then added to each well and incubated at 37°C for 4 h. Subsequently, complete growth DMEM (1 ml) was added to the mixture. Transfected cells were then incubated for 24–72 h in the media (1.3 ml). The cells were then processed to be used in assays.

### Western blot

The cell lysate in RIPA buffer was resuspended with sample loading buffer, boiled for 15 m and centrifuged at 12,000 rpm for 1 m. Total protein (40 to 80 µg) was loaded in each lane, separated by SDS-PAGE gel and transferred onto a PVDF membrane. The membranes were blocked with 3% BSA for biotinylation protein detection or with 3% slim nonfat milk in PBS containing 1.5% Tween 20 and then processed for immunodetection using the following antibodies: Myc, Flag, GFP, HCV NS3, and HRP conjugated anti-mouse or anti-rabbit secondary antibody (CWBio) or using SA-HRP for the detection of biotinylation protein. Protein bands were visualized by adding HRP membrane substrate (Millipore) and then scanned using the ChemiDoc XRS System (Bio-Rad Universal Hood II).

### Reporter assay

Cells (2.5×10^5^ per well in 24-well plates) were cotransfected with expression plasmid DNA (0.1–0.3 µg), Fluc reporter plasmid (0.1 µg) and the internal control vector pRL-TK (0.1 µg) using Lipofectamine 2000 transfection reagent according to the manufacturer's protocol. Twenty-four hours post-transfection, the cells were collected, lysed with 50 µl 1x passive lysis buffer and then subsequently assayed for luciferase activity in cell lysate using the Dual Luciferase Reporter Assay System (Promega).

### Biotinylated protein pull-down assay

HEK293 cells were collected from 6-well plates at 48–72 h post-transfection, washed twice with phosphate-buffered saline (PBS) to remove D-Biotin in the media and then lysed with 60–200 µl RIPA buffer (150 mM NaCl, 50 mM Tris, pH 8.0, 1 mM EDTA, 1% Nonidet P-40, 0.1% SDS, 8340 protease inhibitors (Sigma, 1∶1000)). Approximately 3/4 of the cell lysate was incubated with SA-PMPs (100 to 200 µl) at 4°C for 1 h for binding, followed by 3 washes with RIPA, RIPA high-salt buffer (RIPA buffer with 500 mM NaCl) and RIPA buffer at room temperature. The protein bound to the beads was eluted by boiling the beads for 15 min in RIPA buffer with 1x sample loading buffer. The eluted protein was analyzed by Western blot.

### DLR-PD

HEK293 cells were transfected with HAVI-Fluc tagged (100 ng) and Rluc tagged (100 ng) protein expression vectors in 24-well plates using the Lipofectamine 2000 transfection reagent. At 24–48 h post-transfection, cells were collected, washed twice with PBS to remove D-Biotin from the media and then lysed with 30 µl of 1 x passive lysis buffer (Promega). The Fluc and Rluc activities in the cell lysate (2 µl) in PBS (50 µl) was measured using the DLR assay system before pull-down. Prior to binding, SA-PMPs (20 µl) were washed twice with PBS and resuspended in PBS buffer (50 µl). Then, SA-PMPs were incubated with 2 to 5 µl of cell lysate (more than 1.0E+06 relative light unit) in a 96-well white opaque plate for 30 m to 1 h for binding at room temperature, followed by two washes in PBS with 1% Nonidet P 40 and once with PBS buffer on a magnetic stack. The supernatant of the beads was transferred to another well, and SA-PMP beads were resuspended in PBS (50 µl) for the DLR assay. The dual luciferase activity in the SA-PMP beads, the supernatant of the beads and the cell lysate prior to the binding of SA-PMPs was measured with the Dual Luciferase Reporter Assay System using a Glomax 96 microplate luminometer (Promega). The Rluc activity of beads bound to Fluc was directly measured on the beads to determine the amounts of interacting proteins that bound to the beads after pull down. The percentage of Fluc and Rluc activity on the beads compared with the cell lysate was calculated by dividing the values for the Fluc and Rluc activity on the beads by the Fluc and Rluc activity in the cell lysate. This percentage was used to identify the PPIs. The relative amount of interacting proteins binding to the beads was indicated as the Rluc/Fluc ratio on the beads, which was calculated by dividing the value of Rluc activity on the beads by the value of Fluc activity on the beads. The Rluc/Fluc ratio on the beads was used to analyze the PPIs.

### Statistical analysis

The data represent one of at least two independent assays. All of the results are expressed as the mean and standard deviation of triplicate or duplicate measurements. Student's *t*-test was used to analyze the data.
